# Site Application of Thermally Conductive Concrete Pavement: A Comparison of Its Thermal Effectiveness with Normal Concrete Pavement

**DOI:** 10.3390/ma18153444

**Published:** 2025-07-23

**Authors:** Joo-Young Kim, Jae-Suk Ryou

**Affiliations:** 1 HL Halla Corporation, 81, Pulmugol-ro, Paju-si 10849, Republic of Korea; plus@halla.com; 2 Civil and Environmental Engineering Department, Hanyang University, Jaesung Civil Engineering Building, 222, Wangsimni-ro, Seongdong-gu, Seoul 04763, Republic of Korea

**Keywords:** concrete, pavement, thermal conductivity, icing/snow prevention, wireless data monitoring

## Abstract

In this study, the thermal effectiveness of thermally conductive concrete pavements (TCPs) using silicon carbide (SiC) as a fine aggregate replacement was investigated, compared with that of ordinary Portland cement pavements (OPCPs). The most important purpose of this study is to improve the thermal performance of concrete pavement. Additionally, this study utilized improved thermal properties to enhance the efficiency of pavement heating to prevent icing and snow stacking. Both mixtures met the Korean standards for air content (4.5–6%) and slump (80–150 mm), demonstrating adequate workability. TCP exhibited a higher mechanical performance, with average compressive and flexural strengths of 42.88 MPa and 7.35 MPa, respectively, exceeding the required targets of a 30 MPa compressive strength and a 4.5 MPa flexural strength. The improved strength was mainly attributed to the filler effect and partly due to the van der Waals interactions of the SiC particles. Thermal conductivity tests showed a significant improvement in the TCP (3.20 W/mK), which was approximately twice that of OPCP (1.59 W/mK), indicating an enhanced heat transfer efficiency. In winter field tests, TCP effectively maintained high surface temperatures, overcoming heat loss and outperforming the OPCP. In the site experiment, thermal efficiency was clearly shown in the temperature at the center of the TCP, which was 3.5 °C higher than at the center of the OPCP at the coldest time. These improvements suggest that SiC-reinforced concrete pavements can be practically utilized for effective snow removal and ice mitigation in road systems.

## 1. Introduction

In regions with distinct seasonal changes, special attention must be paid to road pavement surfaces during winter. Snow accumulation can lead to the formation of ice layers, which often cause vehicles to slip and result in tragic accidents [1]. Thus, people use methods to remove the snow or ice layer such as spraying calcium chloride [2] or sand [3], using wind jet blowers [4] or road brushes [5], and turning heating wires on [6]. It is well known that spraying sand is not an effective way to clearly prevent or remove an ice layer or snow because sand just adds additional friction to the snow/ice layer surface. Wind jet blowers are effective in small areas, and road brushes are as well. In particular, road brushes can remove snow from a smaller area than wind jet blowers. Thus, people usually use calcium chloride because facilities for spraying calcium chloride are well-equipped worldwide, cheap, and fast.

Calcium chloride is effective in removing snow and ice layers on road surfaces; however, it makes concrete pavement weaker. It has been reported that the chloride ion component of concrete faces the durability degradation [7]. The effects of chloride ions on concrete cause cracks, spalling, grid mesh corrosion, and pothole breakouts [8,9]. In particular, potholes are the most dangerous factor because they can cause flat tires and ultimately lead to vehicle accidents. Thus, the use of chloride deicing materials is steadily being taken out of consideration.

The most important point of view in recent times has been maintaining the performance of pavement while removing ice layers or snow effectively. To achieve this, there are effective methods that utilize a thermal temperature to melt snow and ice layers. Many studies have Already been performed using their own methods for improving thermal melting systems and can easily be found in the literature. Liu et al. [10] carried out an experiment in which the snow on concrete pavement in an airport was melted using carbon fiber heating wires. The heating wires were installed on the concrete pavement, and electrical power was applied to the wires. The results showed that the thin snow layer fully disappeared after 90 min of heating. After snow melting, the surface temperature of the concrete pavement remained stable at 2.5 °C. In similar work, Huang et al. [11] performed a snow melting experiment using a mock-up test specimen of concrete pavement with installed carbon heating wires. The experiment used a permeable concrete pavement, thus the heat transfer performance was poor. The results showed that ice layers had fully melted after 6 h from when heat was applied [11]. In another ice-melting experiment, the main set parameter was the heat flux—simply the heating power per square meters [12]. As expected, the higher the heat flux applied, the faster the removal of the ice layer. In other words, high energy is invested, and ice can be removed rapidly [12]. The time taken to fully melt the ice was 3.33 h at 600 W/m^2^ [12].

Not only studies that depend on outsourcing, like heating wire, were carried out; these studies were also approached in terms of the material aspects. Representatively, phase change materials (PCMs) have been utilized. The principle is simple: PCMs change their state in a range of ambient temperatures. When changing the state, energy is circulated. Studies of melting snow/ice using PCMs have utilized this phenomenon. According to Farnam et al. [13], the effect of PCM application could be confirmed. When the pavement was heated by the heating sources, the PCMs changed from solid to liquid. In this state, the PCMs store heat energy and release this heat energy when the heating sources are turned off. Due to the energy release phase, the heating period remained longer than that in conventional concrete pavement [13]. Another representative study utilized PCMs in concrete pavement [14]. Deb et al. [14] applied PCM capsules as a lightweight aggregate in the volume fraction. In short, the PCM capsules substituted the aggregate in concrete pavement. The maximum level of application was 15%, and it reduced the snow melting time by 2.3 h compared to that for control [14]. This means that the application of PCMs is definitely effective for melting snow/ice on the surface of concrete pavements.

However, the applications of normal concrete and PCMs have many limitations. In the case of normal concrete, the main problem is the heat transfer. Usually, conventional cement composites have a thermal conductivity (TC) of approximately 1.2–1.7 W/mK [15]. This range of TC values means poor heat transfer performance. As can be confirmed from the studies introduced, most of the results have indicated long melting times. These long melting times originate from low thermal conductivity [16]. Although normal concrete has sufficient strength, its melting snow/ice efficiency is low due to the low TC of normal concrete. In addition, the low efficiency in thermal properties requires high energy consumption.

In contrast, the utilization of PCMs targets the beneficial use of latent heat. However, the critical drawback associated with incorporating PCMs into concrete is the substantial reduction in concrete strength. For instance, Yoo et al. [17] shows the significant decrease in strength when the PCM aggregate is substituted for the fine aggregate. A similar trend was confirmed in the study conducted by Kim et al. [18]. Given that PCMs are organic materials, their inclusion inevitably results in a decrease in the mechanical strength of concrete. Consequently, PCM-incorporated concrete pavement faces practical limitations for use as a road pavement material, where adequate structural strength is essential. In the opposite way, another approach to enhancing the thermal efficiency of concrete pavement involves incorporating thermally conductive materials. Representative examples include carbon-based materials such as carbon nanotubes, graphene, and carbon black; however, these materials are expensive, making them difficult for practical applications. In addition, since the scale of carbon nanotubes is quite small, Van der Waals forces act on the surface of the raw material [16]. Therefore, additional dispersing work is required, resulting in additional costs. For this reason, simply applicable thermally conductive materials are preferred. Silicon carbide (SiC) is a kind of ceramic carbon-based material [6,15,16] that may offer a viable alternative. In particular, the low purity of SiC (94% purity) can be applied to the road pavement material. Although SiC itself is costly due to its widespread use in semiconductor industries, lower-purity SiC is extensively employed in general industrial applications, such as grinding blades, and is commercially available at approximately USD 7 per kg. The particle size is at a level that can be applied to fine aggregates. Considering the price of aggregates, it is true that even low-purity SiC is expensive. However, SiC is a carbonized material and at the same time a ceramic material, and it has sufficient thermal conductivity and hardness. Hence, it is worth applying to fine aggregate substitution material when a proper substitution ratio is to be applied. In practice, Bae et al. [19] showed that the melting time was faster than for the control specimen when the test specimens contained SiC. Furthermore, the compressive strength and TC of the specimens increased up to a certain substitution ratio [19], and the same trend was confirmed in other studies [16,20]. In this manner, using cases of normal concrete and PCMs, diverse studies to improve the snow/ice-melting performance of concrete pavement have been carried out for public safety. In particular, the slab structure scale of mock-up tests has been used to conduct many studies like the introduced cases.

The validation of snow/ice-melting performance is extremely important. However, the use of SiC has only been highlighted in recent years; therefore, real-scale experiments are very rare. Since the materials applied are different from those in existing studies, sufficient verification experiments are necessary. In particular, experimental data on a real scale are of immense value. Concrete pavement with improved thermal properties by SiC application presents a material that is fully feasible in terms of application difficulty and utilization as an industrial by-product from the semiconductor industry. Therefore, there is an urgent need for research related to SiC application in concrete pavement.

In this study, validation of the thermal properties of two kinds of concrete pavement was conducted. One was a normal concrete (NC) pavement, the other was a thermally conductive concrete (TCC) pavement that incorporates SiC. Specimens were installed at Sejong city in the Republic of Korea, and the data were collected from an online cloud data logger system. The experiment was carried out on 30 January 2024. The goal of this study was to improve the thermal performance of concrete. By improving the thermal properties, this study could develop a snow/icing-preventable concrete pavement. The details are indicated in the further chapters.

## 2. Materials and Experimental Plans

**Figure 1:**
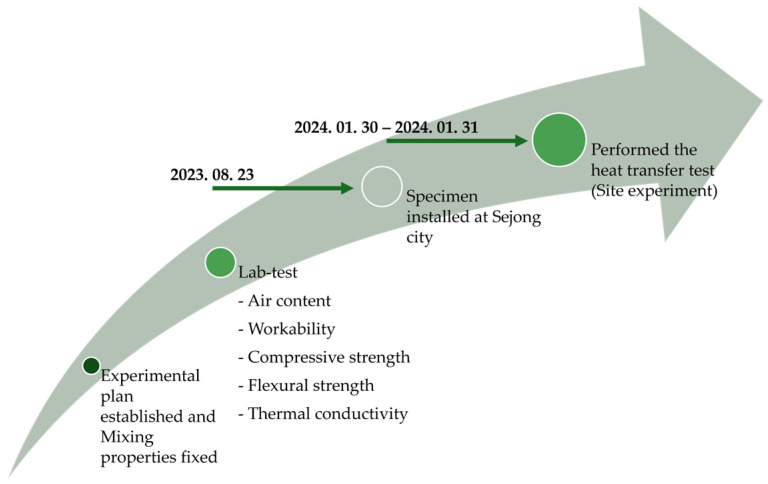
Overall process of this study.

This study includes large-scale, real-world applications. Therefore, the essential factors were thoroughly evaluated beforehand, and the specimens were simultaneously prepared according to strictly controlled procedures. The overall methodology of this study is illustrated in Figure 1.

### 2.1. Specimen Installed Site

**Figure 2:**
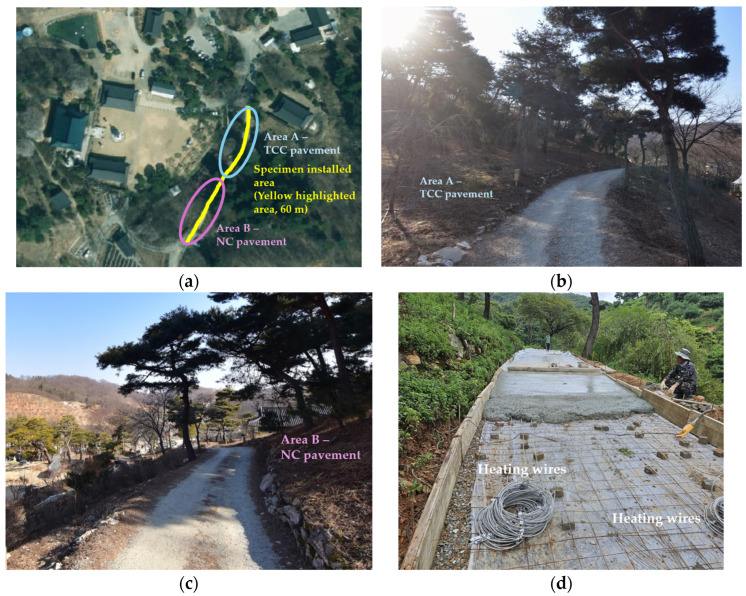

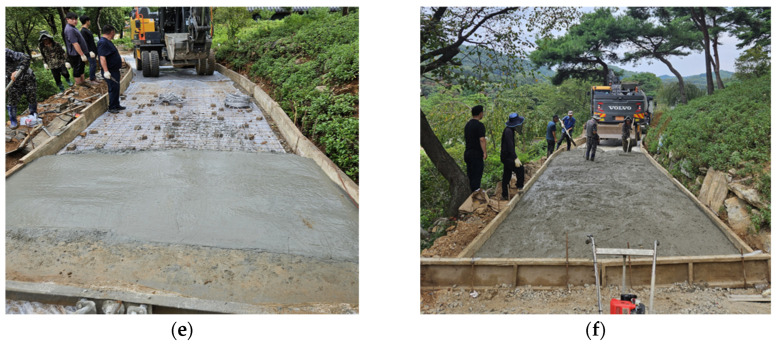
Specimen installation area and casting process: (**a**) satellite view of the experimental area; (**b**) front view of area A; (**c**) front view of area B; (**d**) installing heating wires; (**e**) casting process of TCC; (**f**) casting process of NC.

The site address was Janggun-myeon, Sejong-si, Republic of Korea. The study site was off-road and required concrete pavement coverage. The length of the area to be paved by concrete was 60 m in total; therefore, 30 m was assigned to the NC pavement, and the remaining 30 m was assigned to the TCC pavement. Figure 2 contains the site pictures and specimen installation scene.

The chosen road section exhibits characteristics such as frequent shading and poor meltwater drainage, which tend to prolong icy conditions in winter. Thus, this location is well-suited for validating the heat transfer performance of the TCC compared to NC. As illustrated in Figure 2e,f, due to the narrowness of the installation area, fresh concrete was delivered to the site using a crane. Since January is part of the winter season in the Republic of Korea, continuous vibration compaction was applied to prevent the formation of cold joints during consecutive casting, and each 30 m section was successfully completed.

The specimens were cast on 23 August 2023, and the thermal efficiency experiment was performed from 30 January 2024, 20:13:23 p.m. to 31 January 2024, 13:13:23 p.m.

### 2.2. Cement

The cement used in this study was ordinary Portland cement (OPC), following ASTM C150 [21]. OPC is widely used in the Republic of Korea to make highway road pavement; therefore, this study followed the rules of the material used in the Republic of Korea. According to Table 1, the cement used in this study can be confirmed as OPC with the stated chemical composition. This cement composition has been confirmed in various studies that used OPC [22,23].

### 2.3. Aggregates

Ideally, the specimens should have been produced using natural materials; however, sourcing natural resources proved to be difficult. This shortage of natural aggregates has become a significant challenge in the construction industry in recent years [23]. Hence, there are many methods to be considered to overcome this problem: the use of artificial aggregates [24], recycled aggregates [25], or crushed aggregates [26].

This study decided to use crushed sand and stones as fine aggregate and coarse aggregate because the price was low and they were easy to obtain. Although this study used the crushed aggregates to produce the specimens, the investigation of material properties was performed strictly following ASTM C33 [27].

In the case of fine aggregate, the maximum size was selected as 5 mm, the fineness modulus was 2.83, and the dry density was 2.72 ton/m^3^. In the case of coarse aggregate, the maximum size was selected as 25 mm, the fineness modulus was 7.76, and the dry density was 2.86 ton/m^3^. However, the problem with both aggregates was water absorption. Since these two kinds of aggregates were produced by the crushing method, the matrix of the aggregate itself may have had more voids than natural resources. The water absorption of the fine aggregate appeared to be 3.13%, and 3.22% in the coarse aggregate. The material standard recommends less than 3% water absorption, however, in this case, it was hard to replace the materials. Thus, this study decided to use both aggregates because it was considered that the disparity in values was not that much at only 0.13 and 0.22% over the standard recommendation.

### 2.4. Silicon Carbide and Concrete Mix Properties

SiC was used as a substitution material in the fine aggregate. As mentioned, SiC is a kind of ceramic material; therefore, SiC was appropriate to use as a fine aggregate substitution material. In addition, cases of SiC use is confirmed in many studies [16,20,23]. At a certain substitution ratio, it definitely delivers improvements in thermal/mechanical properties, according to the following studies [16,20,23]. In particular, thermal properties were enhanced by an increase in SiC dosage. This is because SiC has good thermal properties, even though SiC was selected at low purity to make it cost-effective and improve the thermal properties of the concrete pavement. The details of SiC are listed in Table 2. According to Table 2, the maximum particle size of SiC was 120 μm, which makes it unsuitable as a fine aggregate replacement material. However, since the smallest particle size of general fine aggregate is 75 to 150 μm, specific studies have proven that particles within this range can be sufficiently applied as fine aggregate [16,20,23]. Moreover, the smallest particle size of SiC was 40 μm, which contributes to a filler effect in cement composites. Consequently, an improvement in mechanical strength can be expected [28].

Engineering research aims to find the appropriate intersection between the materials dosage and costs. SiC has a higher price than natural/crushed sand; therefore, its cost should be analyzed. To make the specimens, this study had limited a budget, but to improve the thermal properties, the final mix proportions of the specimens were set as in Table 3, using the cost-effective substitution ratio. The names of each specimen are normal concrete pavement for OPCP and thermally conductive concrete pavement for TCP.

To save costs and improve the thermal properties of concrete pavement as much as possible, the conclusion was to set the substitution ratio to 30% for fine aggregate.

### 2.5. Plans for Heating Wires and Sensor Intallation

Installing heating wires inside the concrete matrix incurs additional costs, as does SiC substitution. However, since heating wires were applied simultaneously to OPCP and TCP, this case was assessed as a common expense in this study. As shown in Figure 2d, the carbon heating fibers were covered with high-temperature resistant silicon heating wires and sealed with silver-colored steel-based fiber layers. Thus, this heating wire was bendable, and easy to treat and fix on the wire mesh.

**Figure 3:**
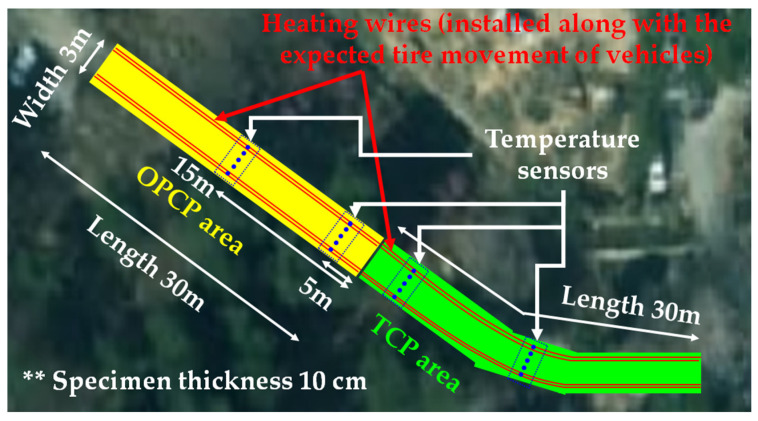
Plans for heating wire installation and sensor attachment.

The heating wire has a diameter of 8.2 mm, a rated voltage of 220 V, a power consumption per hour of 0.9 KWh, and could heat up to 80 °C. The heating wires did not need to be installed over the entire area; therefore, the heating wires were only installed along the area in contact with the tires of moving vehicles. The details of the heating wire plans are indicated in Figure 3.

Figure 3 illustrates the full-scale experimental pavement section designed to evaluate the thermal performance of two types of concrete of OPCP and TCP. The test section is 3 m wide and 60 m long, with each concrete type occupying an equal length of 30 m. The thickness of the specimen was uniformly 10 cm. Heating wires were embedded along the expected tire paths of vehicles to simulate real traffic conditions and provide controlled thermal input, allowing for the assessment of heat transfer performance starting from the heating wires. The total pavement width was 3 m, with a 50 cm gap left on each side. Accordingly, the thermal performance evaluation was conducted within the central 2 m. The heating wires were installed with a spacing of 20 cm, considering the typical width of vehicle tires, and were arranged in pairs on both sides, as shown by the red lines in Figure 3. Given that the specimen thickness was relatively thin at 10 cm, the heating wires were embedded at a depth of 5 cm from the surface to ensure sufficient thermal coverage. Temperature sensors were strategically embedded at multiple locations within both the OPCP and TCP sections to monitor internal temperature variations during heating. Temperature sensors were installed at the starting point, as well as at 5 m and 15 m along the specimen length.. At each point of the temperature sensors, 5 sensors were attached to the surface of the specimens at regular intervals. These sensors enabled a direct comparison of thermal behavior between the two pavement types.

### 2.6. Data Logging System

**Figure 4:**
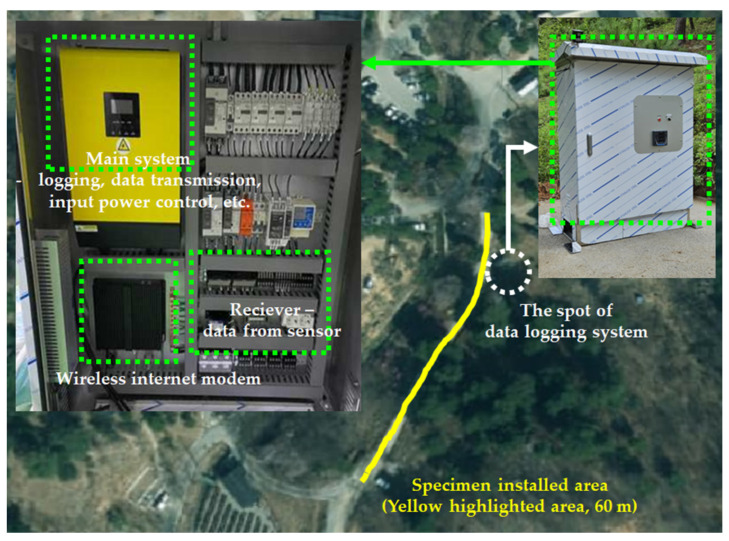
Data logging system.

**Figure 5:**
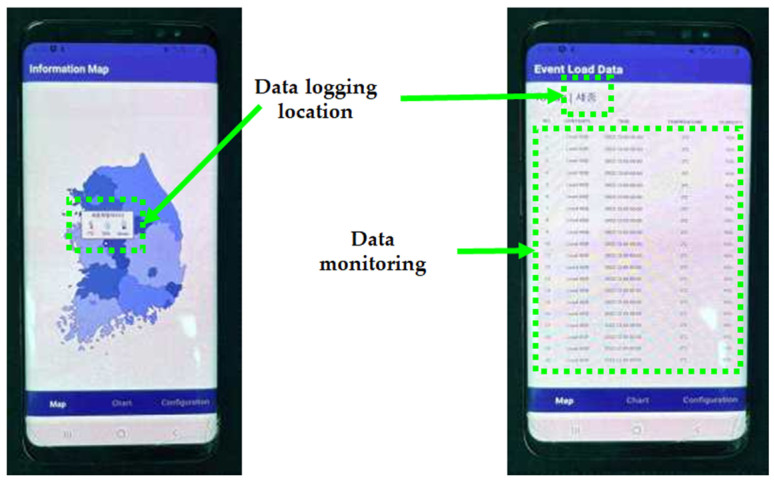
Cloud system mobile application for data logging.

The winter season environment of the Republic of Korea is quite harsh; therefore, most equipment needs to be resistant to cold temperatures and humidity. Experimental equipment and data loggers can retain their functions within an allowable temperature variation. However, the winter in the Republic of Korea is humid and cold; therefore, this study had to find another way to log the data from the sensors. The answer was to use a cloud system to log the sensor data. Figure 4 shows the data logging system used in this study. When the data were transferred to the cloud system, the data could be monitored directly by developed mobile applications. In addition, the data could be downloaded from the cloud in csv file format; therefore, data treatment was comfortable as travel to Sejong city for data collection was not needed. Figure 5 presents a schematic of the cloud-based mobile data logging application.

### 2.7. Experimental Evaluation

#### 2.7.1. Fresh State Evaluation

For concrete pavement, basic specifications should be satisfied. Air content, workability, and compressive/flexural strength are essential assessment components to confirm pavement usability.

The air content of concrete is an essential factor because the Republic of Korea has four seasons, but the temperature variation in the winter season is quite large. Thus, certain values of air content should be satisfied to avoid freeze–thaw damage. The ASTM C231 air content test was followed [29].

Workability is also an important factor. The experimental location was quite narrow, so the delivery time of fresh-state concrete had to be considered. For this reason, sufficient workability was needed. The ASTM C143 slump test was carried out [30].

#### 2.7.2. Compressive and Flexural Strength

Mechanical strength is the most important factor because the minimum level of strength metrics like flexural and compressive strength must be satisfied to ensure service life. According to the pavement design case in the Republic of Korea, it is indicated that concrete should satisfy a flexural strength of 4.5 MPa or more [31]. Usually, flexural strength and compressive strength have a ratio of 1/5–1/7. Following this relationship, the estimated compressive strength should be in the range of 22.5 to 31.5 MPa. Therefore, this study aimed to meet a compressive strength greater than roughly 30 MPa. To assess the compressive (following ASTM C39 [32]) and flexural strength (following ASTM C78 [33]), a universal testing machine (Shimadzu, CCM-200A, Shimadzu Corporation, Tokyo, Japan) was used, which has a capacity of 200 tons of loading. This machine has special equipment for measuring flexural strength; therefore, both properties of compressive and flexural strength could be measured by the same machine. For compressive strength testing, a cylinder-shaped specimen, 100 mm in diameter and 200 mm in height, was utilized, and a beam-shaped specimen of 100×100×400 mm^3^ was applied for the flexural strength test.

#### 2.7.3. Thermal Conductivity Measurement

To evaluate the heat transfer performance of OPCP and TCP, the most important factor is TC. TC is the most exact factor to assess the thermal performance of materials. The TCs of OPCP and TCP were measured using equipment (Applied Precision, isomet-2114, Bratislava, Slovakia). This instrument automatically measures TC 3 times, and the precision of the measurement results is highly reliable [16]. To measure TC, a disk-shaped specimen of ∅100×50 mm^3^ was utilized. The measurement of mechanical strength and TC were lab tests; therefore, the ambient conditions were fixed at 20 °C and 60% relative humidity.

#### 2.7.4. Site Experimental Evaluation of Concrete Pavements Thermal Efficiency

The surface temperatures of the concrete pavements at the pavement heating experiment site were collected. This experiment was performed on one only day, 30 January 2024, because the site was owned by the Korean government and only permitted one day to perform the experiment. The plans are detailed in Section 2.5 and Section 2.6.

## 3. Results and Discussion

### 3.1. Air Content and Workability Test Results

**Figure 6:**
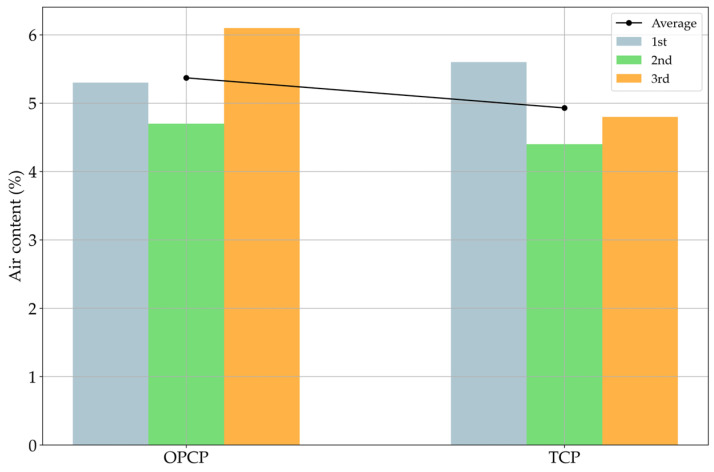
Results of air content tests.

The air content test followed the ASTM method; however, the criterion was based on the Korean air content standard KS F 2409 [34]. The KS standard recommends that the air content of fresh concrete should be in the range of 4.5% to 6%. This entrained air enters the voids inside the concrete matrix, permeating an inducing path of frozen water. The frozen water expands the volume, which causes tensile stress inside the concrete matrix [35]. The fracture mechanism of freeze–thaw damage is repeated tensile stress damage by the expansion of water inside the material [35]. Hence, the voids created by entrained air serve as paths for water expansion, thereby reducing freeze–thaw damage in the concrete [36]. Figure 6 shows the results of the air content test results.

According to the results in Figure 6, the TCP case showed a lower air content value than the OPCP. This phenomenon is related to the filler effect by SiC, and some studies have already discussed it [16,37,38]. In addition, one study replaced 50% of the fine aggregate by weight and reported an increase in compressive strength [37]. This increment in strength highlights the result of the filler effect by SiC substitution [37]. In addition, the typical trend in filler effect reduces the porosity of cement composites; therefore, the results of Figure 6 can be explained by the discussed trends [37,39]. However, the average results of both specimens already satisfied the criteria of Korean air content test standards [34]. Therefore, it was concluded that the mixture properties of concrete could be applied to the experiment site.

**Figure 7:**
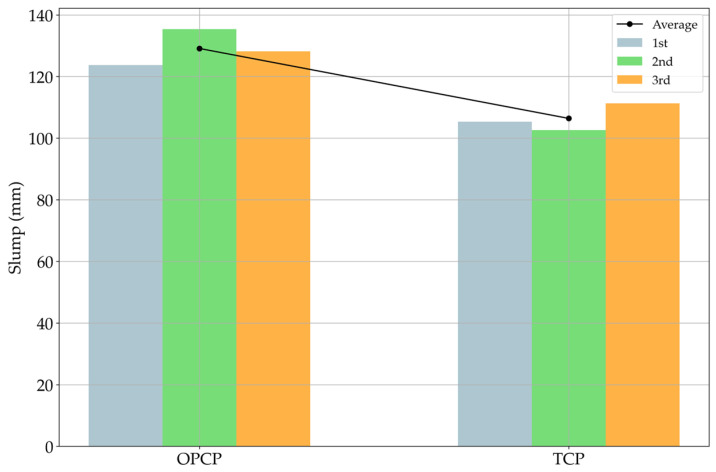
Test results of workability.

The workability results are indicated in Figure 7. Typically, the slump values are applicable to real sites when the values are in the range of 80 mm to 150 mm [40]. According to the results in Figure 7, these mixture properties were considered to be proper mixing contents, as in Figure 6.

Similarly to the decrease in air content due to the filler effect, the TCP also exhibited a lower slump value compared to the OPCP. This phenomenon can be explained by another effect different from the filler effect. As mentioned, SiC has a fine particle size, and the median size is under 100 μm. When matter has a size under 100 μm, van der Waals forces start to strongly affect the surfaces between particles [16]. This could be excusable if just the surfaces between matter particles interacted; however, van der Waals forces affect water as well. This is caused by the molecular structure of water, which shows electrical properties [41]. Due to this reason, SiC particles hold water, which reacts with the cement particles, and this phenomenon reduces the effect of water. This phenomenon can be found described in another study [42]. However, both specimens showed appropriate values for application in the site experiments, so other tests were carried out.

### 3.2. Compressive and Flexural Strength Results

**Figure 8:**
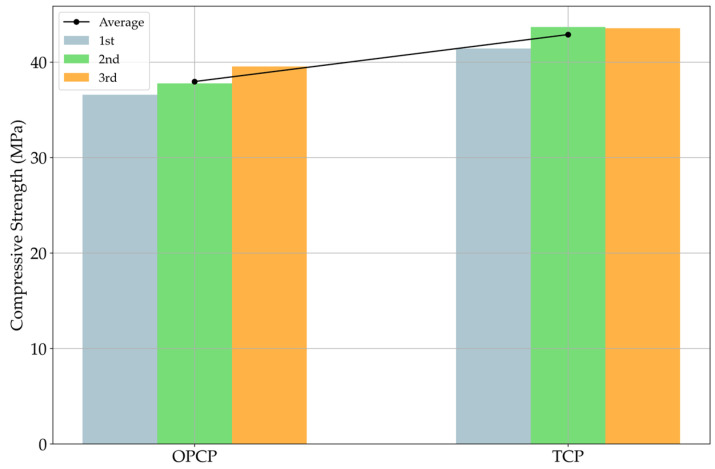
Compressive strength results.

**Figure 9:**
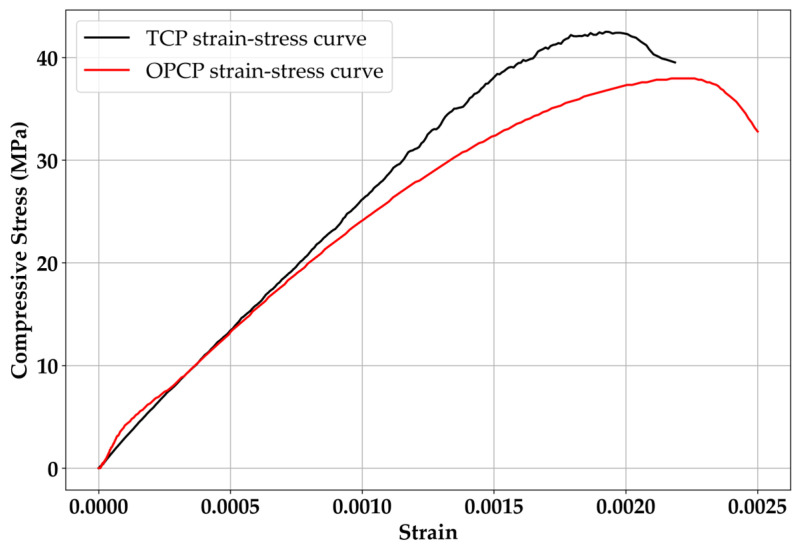
Averaged compressive stress–strain curves of OPCP and TCP.

According to Figure 8, both mixture cases satisfy the target strength of 30 MPa. Not only the individual tests but also the average value of compressive strength is higher than 30 MPa. To more exactly evaluate compressive strength performance, the averaged compressive stress–strain curves of each specimen are indicated in Figure 9.

TCP exhibited higher strength at all test stages as well as in the average value. Further, 30% of SiC substitution for fine aggregate enhances the strength of cement composites normally [16,20,37]. Woo et al. [16] discussed this strength improvement from two perspectives. One is the filler effect, as discussed for air content, as the filler effect usually improves the strength of cement composites [43]. The filler effect makes the matrix of cement composites denser than control specimens, and this phenomenon usually appears when the specimens incorporate the fine particles [43]. Another possible reason is related to van der Waals forces. The dominant factor is absolutely the filler effect. Thus, the effect of van der Waals forces is a minor factor. However, van der Waals force could be discussed with the increment of compressive strength in terms of the electrical interaction of this force [16]. Pulling due to the internal electrical interaction between the hardened cement paste surface and the SiC surface cannot be ignored. According to many studies using nanomaterials [44,45,46], dispersing the nanomaterials before mixing with cement is essential. Because van der Waals forces strongly affect individual particles, agglomeration can be observed [44,45,46]. To disperse nanoparticles efficiently, quite a strong physical force like vibration is needed [47]. From this perspective, although the van der Waals forces are weak at the micro scale, they are stronger on a smaller scale, so van der Waals forces contributed to improving the compressive strength [16].

**Figure 10:**
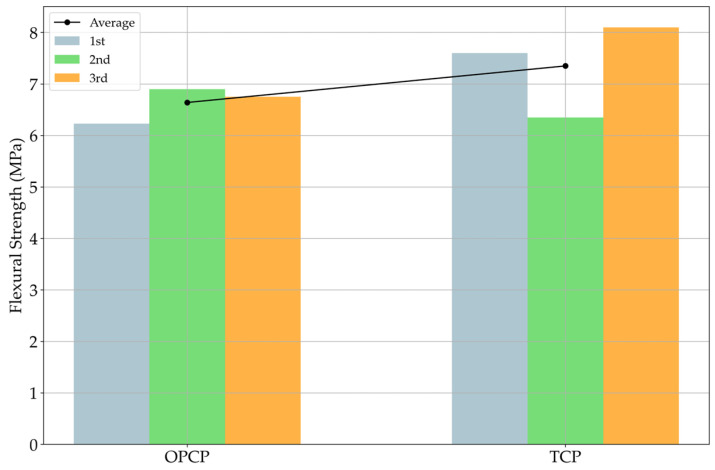
Flexural strength results.

Another test result was the flexural strength test of the OPCP and TCP. Figure 10 summarizes the flexural test results.

The TCP shows a higher flexural strength, like compressive strength. Given the positive relationship between flexural and compressive strength, it is expected that the TCP would exhibit higher flexural strength than OPCP. Notably, both the OPCP and TCP exhibited flexural strengths exceeding the target value of 4.5 MPa specified by the Korean standard [48]. All these tendencies/phenomena can be explained by the same reasons as for compressive strength.

Comprehensively, both mixture properties are appropriate for application to the site experiment.

### 3.3. Thermal Conductivity Measurement Results

**Figure 11:**
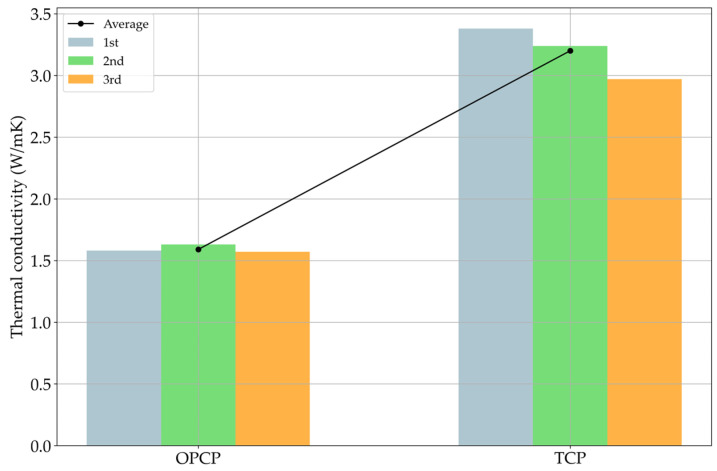
Thermal conductivity results.

The specimens installed in Sejong city were exposed to uncontrolled conditions; therefore, it could not be expected that the TCP would be better than the OPCP without TC values. Since uncontrolled conditions imply the absence of insulation equipment or attachments on the specimens, heat loss could occur at the start of the experiment. Without any TC values, factors like power consumption, operation time, the gap distance between heating wires, and so on could not be decided. Conversely, with TC values, the aforementioned factors could be reasonably determined, which helped to establish the experimental conditions in practice. The TC measurement results are summarized in Figure 11.

The results of Figure 11 show a clear tendency towards improvement in TC when the concrete incorporates SiC. The increase from the OPCP to TCP was approximately twofold, and a similar trend has been reported in prior studies [15,19]. Even when considering uncertainties such as heat loss that may occur in the field, the difference in TC observed in Figure 11 is expected to sufficiently reflect a difference in performance. The TC shown for the cement composites does not mean that the heat transfer efficiency doubled. When comparing existing studies, the heat transfer efficiency indicates that when the TC is doubled, the heat transfer efficiency is improved in the range of 40 to 50% [15,19]. In addition, when heat is transferred to snow or ice, the time at which the efficiency shows changes depends on the target materials [16,19].

By comprehensively reviewing the results for fresh properties, mechanical strength, and TC, the overall plan for the site experiment described in Section 2 was finalized.

### 3.4. Site Experiment for Assessing the Heat Transfer Performance Results

**Figure 12:**
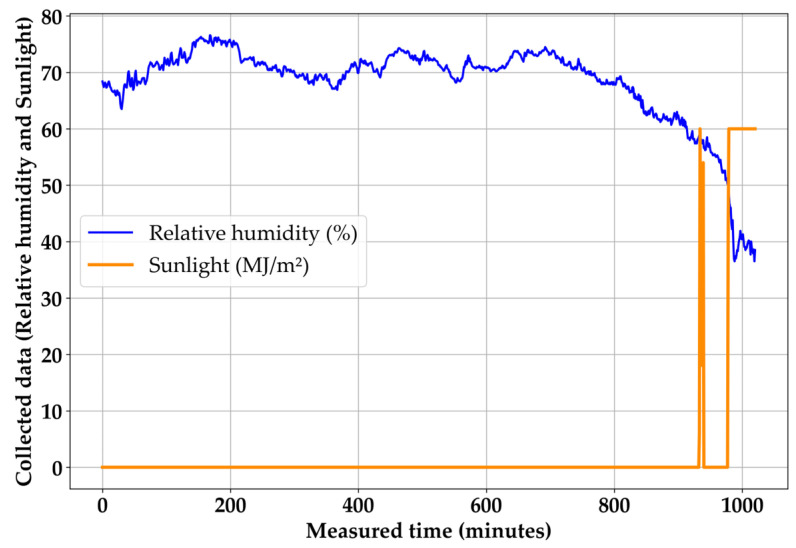
Collected ambient data.

**Figure 13:**
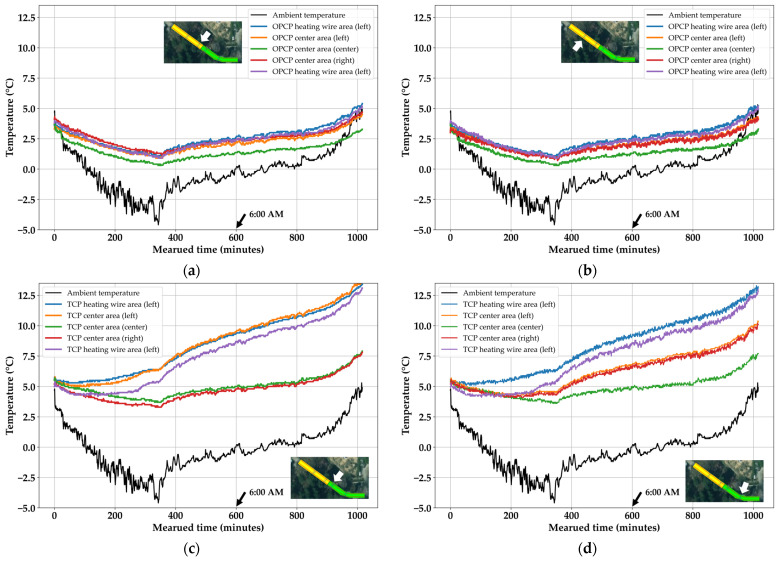
Site experiment results: (**a**) OPCP site 1; (**b**) OPCP site 2; (**c**) TCP site 1; (**d**) TCP site 2.

The heating system was activated on 30 January 2024 at 20:13:23. To evaluate the exact heat transfer performance, the effects of sunlight and relative humidity were examined first, and the results are presented in Figure 12. After evaluating environmental influence, the results of the heat transfer experiments were discussed. As can be seen from the ambient temperature in Figure 13, although the 600 min point was 6:00 a.m., one characteristic of the Republic of Korea in winter could be observed. The winter temperature fluctuates daily, with days with cold temperatures and days with warm temperatures occurring periodically. In particular, the starting point for changing from cold to warm temperatures appears at dawn, and this phenomenon was observed during the experimental period of this study. However, since it was below zero before 6:00 a.m., there was no significant problem in evaluating the heat transfer performance.

Firstly, the influence of environmental factors should be discussed. In Figure 12, the relative humidity and sunlight per minute is confirmed. Compared to the black lines in Figure 13, the relative humidity exhibits behavior opposite to that of ambient temperature, which is a common phenomenon given their inverse relationship. The most important factor in Figure 12 is sunlight. This experiment was completed by noon on 31 January 2024. Thus, it presented a difficult situation for the evaluation of heat transfer performance because the sunlight’s influence was stronger than expected, especially on concrete pavement [6]. However, it can be confirmed that the sunlight did not appear for most of the experiment duration. This indicates that the weather was cloudy; therefore, the sunlight could not directly affect the surface of the specimens. Only the last 80 min are considered to have been under the influence of sunlight. Hence, the heat transfer performance could be evaluated with minimal interference from sunlight.

Based on the sensing results for the two sections of the OPCP, the OPCP was found to be greatly affected by changes in ambient temperature. In other words, it followed the trend in the ambient temperature. The temperature at the center of the road pavement was the lowest, which meant that heat transfer from the heating wire to the center was not performed well. In addition, unlike the TCP, the temperature was significantly lower in the heating wire section and near the heating wire, which confirmed that 5 cm of heating wire installation was fatal to the OPCP. In other words, this implies that heat transfer to the surface was not performed properly, and that heat loss to the surroundings was serious. This phenomenon of the OPCP is indirectly confirmed in other studies. In the study of Woo et al. [16], a normal specimen like OPCP showed the poorest melting performance in an ice-melting experiment. In addition, according to the study of Bae et al. [19], low TC shows seriously poor thermal performance, as in the study of Woo et al. [16]. Bae et al. [19] and Woo et al. [16] found the poorest performance with OPCP, even though the normal specimens prevented heat loss in the test when evaluating pure thermal performance. In other words, it can be said that an improvement in the TC intuitively reflects an improvement in the thermal performance. Most noteworthy is that no heat loss-prevention system was applied to the test object in this study. Compared with the studies of Bae et al. [19] and Woo et al. [16], the results of Figure 13 can be explained. In summary, it was confirmed that temperature transfer to the surface and surroundings was not properly achieved due to the low thermal conductivity of OPCP.

Conversely, it can be seen that for the TCP, the temperature of the hot wire and the surroundings of the hot wire tend to rise steadily, overcoming the ambient temperature. This suggests that the thermal conductivity of 3.20 W/mK exceeded the critical point where heat loss is overcome and heat transfer to the concrete surface and surroundings occurs. The most encouraging result is that the temperature in the center area of the TCP was maintained above 3 and after 6:00 a.m., the temperature increased along with the trend in the hot wire. Overall, the partial substitution of the fine aggregate with SiC increased the thermal conductivity, which potentially implies an improvement in the snow/ice-melting performance. Compared simply to prior studies using SiC [16,19], the largest decrement in ice-melting time was seen with 30% SiC substitution of fine aggregate. The studies showed a 14-min decrease in the average ice-melting time with 0 to 30% substitution [16,19]. However, for the results of 30% to 50% substitution, the decrement of ice-melting time was an 8 min on average [16,19]. It can be concluded that the threshold of improvement in specific thermal performance was exceeded due to a SiC substitution of 30%. In addition, the results of these two studies [16,19] suggest that the result shows a continuous temperature increase without preventing heat loss in this study. This also indicates that if the system is operated in advance in conjunction with the weather system, the road surface can be maintained as usual.

## 4. Conclusions

This study investigated the heat transfer performance of thermal performance-enhanced concrete. The TCP incorporated SiC as a substitute material for fine aggregate. Due to the application of SiC, the TCP showed many improvements compared to OPCP. The comprehensive results are as follows:

1. The experimental results show that both the OPCP and TCP mixtures met the Korean air content and workability standards. The TCP showed a slightly reduced air content and slump value due to the filler effect of SiC and the fine particle size. The filler effect reduced the porosity and contributed to the improvement in the compressive strength. The van der Waals interactions of SiC particles smaller than 100 μm induced local moisture retention, which affected the effective water utilization rate. Despite these changes, both mixtures showed properties suitable for practical field applications.
2. Both the OPCP and TCP mixtures exceeded the compressive and flexural strength targets required by the Korean standard. The TCP showed higher mechanical performance due to the densification of the cement matrix due to the filler effect of fine SiC particles. In addition, van der Waals interactions contributed to the strength improvement through electrical interactions and particle dispersion behavior. Overall, the mechanical properties of both mixtures are suitable for field applications.
3. The TCP showed approximately twice the thermal conductivity of OPCP (3.20 W/mK), effectively overcoming heat loss and enabling surface heat transfer. In the site experiment, TCP maintained higher temperatures at the center and heating wire zones, proving superior thermal performance. In contrast, the OPCP exhibited poor heat transfer and significant heat loss due to its low thermal conductivity. Therefore, partial substitution of SiC can enhance snow- and ice-melting performance, offering a practical solution for winter pavement systems.
4. This experimental system is a prototype of a snow/ice-prevention system for concrete pavement. It is not a final version of the experiment; however, one aspect of heat transfer performance improvement was confirmed. As this technology is enhanced, a total solution of a snow/ice-prevention system containing wireless cloud system data logging will be completed.

## Figures and Tables

**Table 1:** Properties of OPC.

Chemical Composition (%)						Density (ton/m^3^)
CaO	SiO_2_	Al_2_O_3_	Fe_2_O_3_	MgO	SO_3_	
62.7	21.5	6.3	4.2	3.2	2.3	3.153

**Table 2:** SiC properties.

Chemical Composition (%)				
Density (ton/m^3^)	Maximum Particle Size (μm)	Elastic Modulus (GPa)	Thermal Conductivity (W/mK)	Purity of SiC (%)
3.2	120	193	25.5	94

**Table 3:** Mixture properties.

Specimens	Mix Properties (kg/m^3^)					
	Water	Cement	Fine Agg	Coarse Agg	SiC	Water Reducer
OPCP	230	460	816	1073	0	4.6
TCP			571		245	

## Data Availability

The original contributions presented in this study are included in the article. Further inquiries can be directed to the corresponding author.
